# Ageing and its implications

**DOI:** 10.4103/0973-029X.72500

**Published:** 2010

**Authors:** P Jayanthi, Elizabeth Joshua, K Ranganathan

**Affiliations:** *Department of Oral and Maxillofacial Pathology, Ragas Dental College and Hospital, Chennai, India*

**Keywords:** Ageing, progeroid, telomerase, telomere, theories

## Abstract

Ageing processes are defined as those that increase the susceptibility of individuals, as they grow older, to the factors that eventually lead to death. It is a complex multi-factorial process, where several factors may interact simultaneously and may operate at many levels of functional organization. The heterogeneity of ageing phenotype among individuals of the same species and differences in longevity among species are due to the contribution of both genetic and environmental factors in shaping the life span. The various theories of ageing and their proposed roles are discussed in this review.

## INTRODUCTION

For instance, individuals with a deleterious Ageing is a progressive, deleterious and intrinsic phenomenon in an organism. In 1952, the process of ageing was referred to by Medawar as “unsolved problem in biology.” Although the ageing process still remains a biologic mystery, research has been ongoing since Medawar’s time and thus, knowledge in this aspect is expanding rapidly.[1]

Two fundamental parameters of ageing are average and maximum life span. The average life span, also known as life expectancy, is represented by the age at which 50% of a given population survives, while the maximum life span represents the longest-lived member of the population.[1] An increased interest in the study of ageing in recent times has been stimulated by lengthening of the average human life span, lengthening of the maximum human life span, increased percentage of elderly in the population and increased use of national economic resources by the geriatric population.[2]

Ageing and senescence are related words and are often used interchangeably as both processes are characterized by progressive changes in the tissue of the body, eventually leading to a decline in function and death of the organism. Senescence refers to a post-maturational process that leads to diminished homeostasis and increased vulnerability of the organism to death. Ageing, in contrast, refers to any time-related process and is a continuous process that starts at conception and continues until death.[1] The mechanisms involved in ageing are partially intrinsic to the organism, like genetic and epigenetic factors, and partially to the external origin, such as nutrition, radiation, temperature and stress.[3]

## THEORIES OF AGEING

Various theories have evolved to improve our understanding of the ageing process so as to formulate strategies that enhance extension of life. The theories of ageing are classified based on the level at which the ageing mechanism is targeted, as:[4]

1. Evolutionary theories
2. Systemic theories
3. Molecular and cellular theories

### Evolutionary theories

Evolutionary theories state that ageing results from a decline in the force of natural selection. As evolution acts primarily to maximize reproductive fitness in an individual, longevity is a trait to be selected only if it is beneficial for fitness. Life span is, therefore, the result of selective pressures and may have a large degree of plasticity within an individual species as well as among species.[2] The various evolutionary theories are:

#### Mutation accumulation theory

This evolutionary theory suggested by Peter Medawar states that detrimental late-acting mutations may accumulate in the population that ultimately leads to pathology and senescence. According to Medawar, ageing is a non-adaptive trait. If some function cannot be used to provide reproductive advantage, it will not be supported by selective pressure. These functions are lost in future generations.

For instance, individuals with a deleterious mutation have fewer chances to reproduce if the deleterious effect is expressed earlier in life. The condition that exemplifies this theory is Progeria. Progeroid individuals exhibit premature ageing signs, live shorter life spans and are less likely to pass their mutant genes to subsequent generations.

For instance, individuals with a deleterious mutation have fewer chances to reproduce if the deleterious effect is expressed earlier in life. The condition that exemplifies this theory is Progeria. Progeroid individuals exhibit premature ageing signs, live shorter life spans and are less likely to pass their mutant genes to subsequent generations.[2,5]

#### Disposable soma theory

According to this theory, the somatic organism is effectively maintained only for reproductive success and, afterwards, it is disposable due to lack of resources. This theory proposes that the resources in the body are invested toward reproductive success. Somatic maintenance or longevity is related to the balance or imbalances in resources set aside for reproduction. This theory explains why we live for a specific length of time but does not explain any specific cause for ageing.[2,4,5]

#### Antagonistic pleiotropy theory

The theory of antagonistic pleiotropy suggests that some genes may be selected for beneficial effects early in life, yet have unselected deleterious effects with age, thereby contributing to senescence. For example, mutations causing overproduction of sex hormones in young age may increase the sex drive, libido and reproductive success, while in older age, the same hormones lead to prostrate cancer in males.[2,4,5]

### Systemic theories

In systemic theories, the ageing process is related to the decline of organ systems essential for control and maintenance of other systems within the organism.

#### Neuroendocrine theory

This theory proposes that ageing is due to changes in neural and endocrine functions that are crucial for coordinating communication and responsiveness of all body systems with the external environment. An important component of this theory is the role of the hypothalamus–pituitary axis (HPA). It has been suggested that the decreased ability to survive stress in old age may be due to a failing HPA. However, many organisms with an ageing phenotype similar to that of higher vertebrates lack complex neuroendocrine systems. The changes that occur in the neuroendocrine system may be due to fundamental age-related changes in all cells and may therefore be secondary manifestations of the ageing phenotype.[2,4]

#### Immunologic theory

This theory was proposed by Walford in 1969. It was hypothesized that the normal process of ageing in man and in all animals is related to a faulty immune processes. The immunologic theory of ageing is based on two main observations:

- The functional capacity of the immune system declines with age, as evidenced by a decreased response of T cells to mitogens and reduced resistance to infectious disease
- Autoimmunity phenomena increase with age[6]

### Molecular and cellular theories

These theories attempt to discern the mechanisms of ageing process at the cellular and subcellular levels.

#### Error catastrophic theory

This theory was proposed by Leslie Orgel in 1963 and is also called as “Orgel’s hypothesis.” The hypothesis stated that if an error is made in the molecular copying processes (transcription or translation), it would result in flawed synthesis of a given protein. It has been observed that damaged proteins accumulate with age. This can lead to cellular dysfunction and accumulation of other forms of damage and senescence. But, in experiments, when abnormal amino acids were fed to animals, they failed to result in a shorter life span. Hence, Orgel’s hypothesis has not been accepted.[2,7]

#### Free radical theory

In 1956, Denham Harman suggested that free radicals produced during aerobic respiration cause cumulative oxidative damage, resulting in ageing and death. The targets of these free radicals are lipids, nucleic acids and proteins, leading to autoxidation of lipids, cross-linking of proteins and nucleic acids, altered cell membrane properties and peptide fragmentation.[8]

Most experimental evidence in favor of the free radical theory of ageing comes from invertebrates. In a study by Schriner *et al*.,[9] it was observed that mice with extra catalase in their mitochondria lived 18% more than controls and were less likely to develop cataracts, but they did not appear to age slower.

The free radical theory appears to be one of the most promising explanations for the process of ageing. But, numerous experiments have shown contrasting results. Also, the prime cause for opposition to this theory arises from the fact that antioxidant supplementation has not shown to decelerate ageing.

#### Waste product theory

It is hypothesized that age-related accumulation or modification of certain substances in post-mitotic cells may play a role in ageing.[4]

Many substances have been identified that act as biomarkers of ageing, such as:

### a) Lipofuscin[2,4]

The gradual and steady accumulation of intracellular yellow–brown fluorescent pigment, referred to as lipofuscin, occurs in numerous organisms. Although it is continuously produced, it gets diluted by successive rounds of cell division among daughter cells. Mazabradi *et al*. (1988, 1995), in their experiments using cultured rat myocardial cell, demonstrated that

- Lipofuscin may be produced either due to oxidative damage or lysosomal dysfunction
- Lipofuscin is more likely a result of ageing than a cause

### b) Advanced glycation end products (AGE)

Dr. Cerami suggested that non-enzymatic reactions of glucose and other reducing sugars with amino groups of proteins and nucleic acids result in a series of events that alter the protein and nucleic acid structure and function. It is still not clear whether these proteins are physiologically relevant or whether they serve as mere markers.

Glucose reacts with amino groups in proteins to form Schiff bases and Amadori products. The Amadori products break down to form reactive dicarbonyl glyoxal compounds (AGE) like 3-deoxyglucosome, methylglyoxal and glyoxal. This process is described as Maillard reaction. AGE usually affect long-lived stable proteins by causing cross-links. Collagen, myelin, fibrinogen, tubulin and plasminogen activator are usually affected, with collagen being the prime target.[10]

Recently, a specific receptor for AGE, called Receptor of Advanced Glycation End Products (RAGE), has been identified on mononuclear cells and endothelial cells. One of the effects of AGE binding by RAGE is generation of intracellular oxidants, activation of transcription factor NF-кB and induction of downstream events linked to atherogenesis. The RAGE binding of AGE results in generation of free radicals and has been implicated in Alzheimer’s disease.[10,11]

#### DNA damage in ageing

Leo Szilard in 1959 first proposed that accumulation of DNA damage causes ageing. Progeroid syndromes or premature ageing syndromes are cited as examples to elucidate the role of DNA damage in ageing. The progeroid syndromes, Werner’s syndrome (WS), Hutchinson-Gilford’s syndrome and Cockayne syndrome, originate in genes that are related to DNA repair/metabolism.[2,4]

WS originates in a recessive mutation in the WRN gene, which encodes a helicase and also possesses an exonuclease activity. This suggests that WRN may be involved in DNA repair. It has been observed that cells taken from patients with WS have increased genomic instability. Thus, it has been proposed that the WS model is an indicator that alterations in DNA play a role in ageing.

Hutchinson-Gilford syndrome is caused by a mutation in genes that code for the nuclear protein lamin A. The exact functions of lamin A remain unknown, but it may be involved in the functioning of the inner nuclear membrane. Further evidence suggests that the DNA machinery is impaired in this syndrome, reinforcing the idea that the changes in DNA are important in these diseases and, thereby, in normal ageing.[12]

The protein involved in the Cockayne syndrome participates in transcription and DNA metabolism. If Progeroid syndromes represent a phenotype of accelerated ageing, then changes in DNA are likely to play a role in ageing. But, it is argued that many genetic disturbances affecting DNA repair do not influence ageing. Thus, it is doubtful whether overall DNA repair is related to ageing or DNA damage accumulation alone drives ageing.[12]

The protein involved in the Cockayne syndrome participates in transcription and DNA metabolism. If Progeroid syndromes represent a phenotype of accelerated ageing, then changes in DNA are likely to play a role in ageing. But, it is argued that many genetic disturbances affecting DNA repair do not influence ageing. Thus, it is doubtful whether overall DNA repair is related to ageing or DNA damage accumulation alone drives ageing.[12]

#### Telomere theory

Telomeres are physical ends of linear eukaryotic chromosomes whose functions are protection, replication and stabilization of chromosome ends. Telomeres contain stretches of tandemly repeated DNA sequences composed of a G-rich strand and a C-rich strand (terminal repeats). These terminal repeats are highly conserved among vertebrates, which appear to have the same sequence repeat (TTAGGG)n.

Telomeres were implicated in the ageing process for the first time by Watson in 1972. Cells with short telomeres did not divide. Once a critical length of telomeres was reached, the cells became senescent; this is called Hayflick’s limit. The Hayflick’s limit represents permanent growth arrest. This was the foundation of the telomere hypothesis of ageing.[13]

Telomerase is a reverse transcriptase enzyme that adds hexameric repeats, TTAGGG, to chromosome ends, extending and maintaining the length of telomeres and, thereby, extending the number of divisions the cell may undergo. Blackburn and Greider identified the enzyme in the nematode *C. elegans*. The introduction of the catalytic protein (hTERT) component of telomerase into normal fibroblasts and epithelial cells prevents shortening of the telomeres and results in immortalization.[14]

The evidence favoring the telomere hypothesis of ageing is:[10]

- Telomeres from cells of older individuals are shorter than those from cells of younger individuals.
- Telomeres are shorter in germ line cells than in somatic cells.
- Experimental elongation of telomeres prolongs the proliferative capacity of normal cells and cells from Progeroid patients.

Telomere dynamics have been measured for a number of Progeroid syndromes. For both ataxia telengiectasia and Hutchinson-Gilford progeria, telomeres are shorter than that for age-matched normal controls. Orren *et al*.^15^ have demonstrated that the mutated WRN gene in WS binds to the D-loop of the telomere and disrupts the telomere structure.

Dyskeratosis congenita (DKC) is an X-linked disorder characterized by skin and bone marrow pathologies. Intraorally, the tongue and buccal mucosa develop bullae. These are followed by erosions and, eventually, leukoplakic lesions. The leukoplakic lesions are considered to be premalignant and approximately one-third of them become malignant in a 10–30-year period. The mutation responsible for DKC affects an enzyme involved in the metabolism of the telomerase RNA subunit (hTR). A rare dominant autosomal form of DKC can result from mutation of the hTR gene directly, supporting the idea that DKC manifests itself through telomerase dysfunction. The increased incidence of carcinomas in DKC patients suggest that telomere shortening may contribute to the development of cancer that is more prevalent with age.[2]

The caveat is that telomere–telomerase-deficient mice have not shown all the phenotypes of ageing. Also, mere reexpression of telomerase does not always readily induce lengthening of telomeres and reversal of senescent phenotype. Thus, although telomeres are likely to play a profound role in ageing, there may exist multiple levels of regulation of telomeres and telomerase in ageing.[12]

## CONCLUSION

Many separate mechanisms that potentially affect ageing have been recognized, such as:

- Multiple pathways of DNA repair
- Defenses against free radicals such as antioxidants and enzymes
- Removal of abnormal proteins and prevention of their accumulation or formation
- Immune response
- Cellular replicative senescence and apoptosis

Although some headway has been made in solving the “riddle of the ages,” we are still no closer to the end of the tunnel that explains the process of ageing and the means to effectively countering it. While attainment of immortality is an impossible goal, future research is needed into the benefits of hormonal and genetic manipulation as successful interventions to improve longevity and quality of life.
